# Anatomical evaluation of the transpubic screw corridor based on a 3D statistical model of the pelvic ring

**DOI:** 10.1038/s41598-021-96219-5

**Published:** 2021-08-17

**Authors:** Charlotte Arand, Daniel Wagner, Robert Geoff Richards, Hansrudi Noser, Lukas Kamer, Dominic Gehweiler, Johannes Hopf, Pol M. Rommens

**Affiliations:** 1 Department of Orthopaedics and Traumatology, University Medical Center, Langenbeckstraße 1, 55131 Mainz, Germany; 2 AO Research Institut, Clavadelerstrasse 8, 7270 Davos, Switzerland

**Keywords:** Anatomy, Musculoskeletal system, Bone

## Abstract

Retrograde transpubic screw fixation is a common procedure for the treatment of anterior pelvic ring fractures. With its sparing surgical approach and significant pain relief after screw fixations allowing early mobilisation, it has gained importance especially in the treatment of insufficiency fractures in elderly patients. However, positioning of transpubic screw osteosynthesis is not always possible due to narrowness and curvature of the screw corridor. The aim of the present study was to evaluate availability and length of the screw corridor using a 3D statistical model of the pelvic ring consisting out of 150 uninjured pelves. Virtual bore probes with a diameter of 7.5 mm were analysed as to accessibility, length and grey value distribution in Hounsfield Unit (HU). A transpubic corridor with a diameter of ≥ 7.5 mm was available in 185 of 300 investigated superior pubic rami with mean screw length of 131.7 mm. Accessibility of the screw corridor was higher in males than in females. However, screw length showed no systematic differences between the sexes or ethnicities. Analysis of the grey value distribution demonstrated the strongest bone to be located at the lateral ilium and the supraacetabular region.

## Introduction

Retrograde transpubic screw fixation is a frequently used surgical procedure for the treatment of fractures of the anterior pelvic ring^1–4^. It is a minimally invasive procedure which can be used in minimally or non-displaced fractures of the superior pubic ramus. In cases with displacement of fracture fragments, retrograde screw osteosynthesis can be performed after closed or open reduction^5^. Alternatively, a reduction manoeuvre utilizing a provisional screw can be used^6^. The latter method has gained importance especially in the treatment of geriatric patients with fragility fractures of the pelvic ring due to its sparing surgical approach, significant pain reduction after stabilisation and subsequent early mobilisation of the patient^7–11^. As the number of geriatric fractures increases, percutaneous fixation methods become more relevant^12–15^.

Several biomechanical studies have investigated the stability of screw osteosynthesis of the anterior pelvic ring^16,17^. Various clinical studies and case series have demonstrated low complication rates and good to excellent clinical results^1^. However, some concerns regarding this method are remaining. Positioning of the retrograde transpubic screw is not always possible without affecting the acetabular joint surface due to the curvature of the screw corridor. Proximity of crucial neurovascular structures such as the ilioinguinal and genitofemoral nerves and the obturator and the external pudendal artery around the entry point, as well as the external iliac vessels passing just proximal the superior pubic ramus with direct contact to the bone, can be critical and limiting for the procedure^18^. Further, not in every patient a screw with an adequate diameter can be placed due to narrowness of the screw corridor^19–21^. In addition, screw loosening, backing out and pseudarthrosis are described as potential complications during follow-up^1,22,23^.

**Figure 1:**
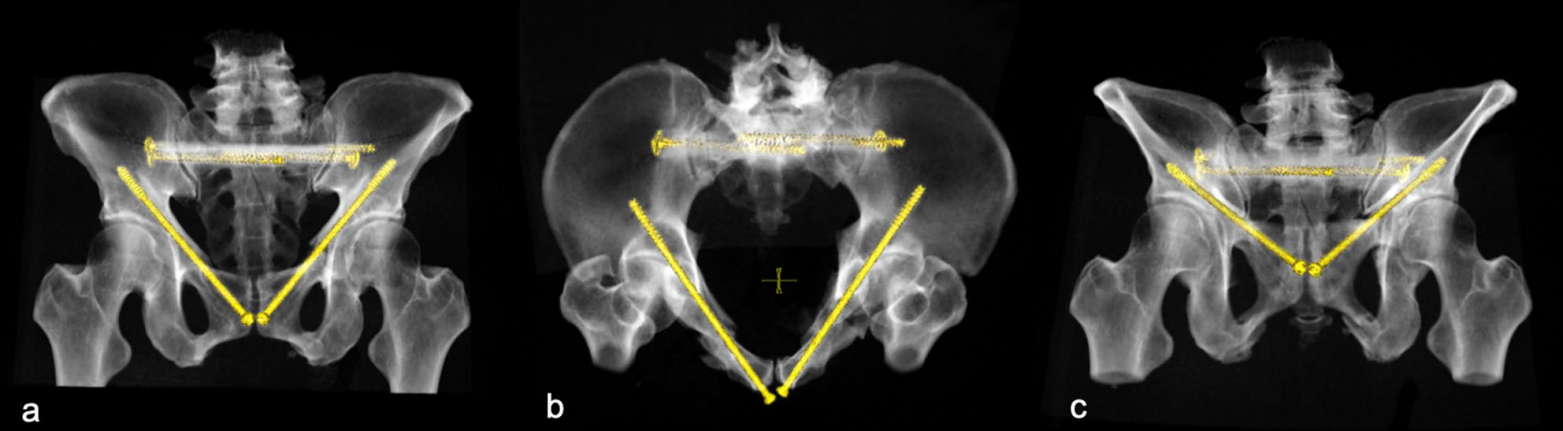
Bilateral retrograde transpubic screw fixation in a patient pelvic ring fracture after traffic accident **(a)** ap view **(b)** inlet view **(c)** outlet view. This figure was generated using the software Sectra PACS (Version 22.1, Linköping, Sweden; www.medical.sectra.com).

The aim of the present study was the evaluation of the potential screw corridor for positioning a screw with a diameter of 7.3 mm, as well as the analysis of the local bone stock within the screw corridor. Figure 1 shows the status after bilateral retrograde transpubic screw fixation of bilateral superior pubic rami fractures and an additional posterior fixation in a trauma patient with pelvic ring fracture.

## Material and methods

Analysis was performed based on a 3D statistical model of the pelvic ring comprising of 150 CT scans of uninjured adults (100 Europeans: 51 male, 49 female, average age 60.0  ± 13.1 years; 50 Japanese: 30 male, 20 female, average age 74.3  ± 17.5 years). Average image resolution of the raw data was 0.7 × 0.7 × 0.6 mm. CT scans had been acquired for indications not related to the musculoskeletal system prior to the start of the study. The study was approved by the local ethics committee (Ethikkommission Bundesland Salzburg, Austria). Informed consent was obtained from all patients, data acquisition, processing and analysis were in accordance with all legal regulations and guidelines.

The 3D statistical model was computed using the software Amira (Amira version 6.0.0, FEI, Hillsboro, OR, USA) as previously described by our group^24^. The usage of a statistical model, consisting out of more than 300.000 homologous surface triangles, allowed for a landmark-based definition of the individual screw corridor.

**Figure 2:**
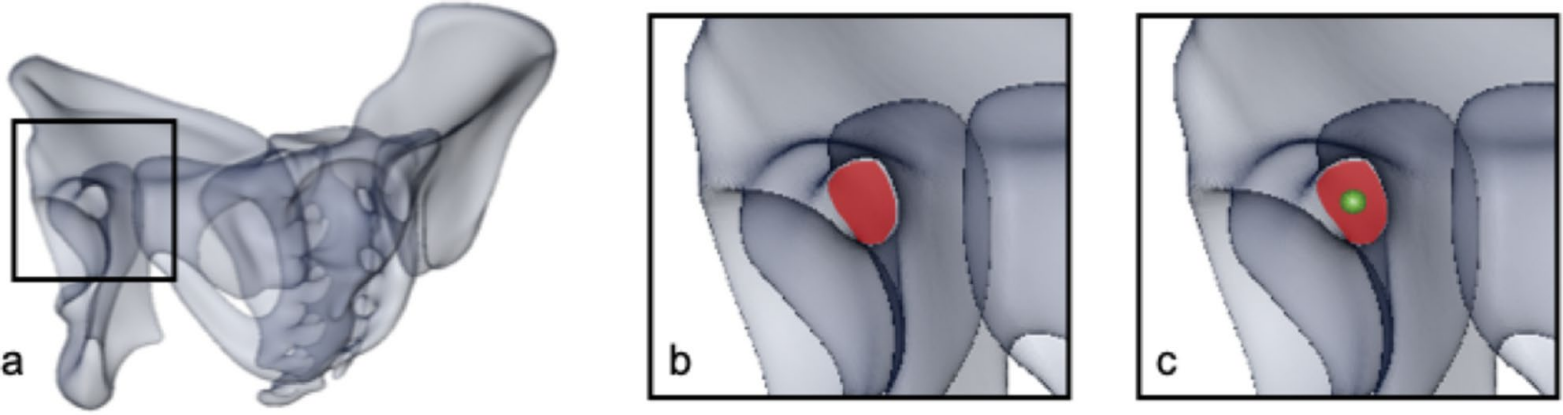
**(a)** Orientation of the 3D model in so-called gun barrel view^25^, **(b)** screw corridor (marked in red), **(c)** marked screw entry point in the center of the corridor (green dot). This figure was generated using the software Amira (Version 6.0.0, FEI, Hillsboro, OR, USA; www.fei.com).

Thus, virtual screw positioning was performed by defining entry and exit point of the screw on the 3D surface models using Amira software. The overall mean model and each of the 150 individual computer models were oriented in a way allowing for a direct view into the screw corridor, described as gun barrel view^25^ (Fig. 2a). In a second step, orientation was adjusted so that the maximum diameter of the screw corridor was displayed (Fig. 2b). The landmarks defining entry and exit point of the virtual screw were positioned in the region of the pubic tubercle and the posterior ilium in the center of the projected corridor (Fig. 2c). Between those two landmarks, a virtual bore probe with a diameter of 7.5 mm was placed in order to simulate screw positioning. Length and local grey values of the virtual bore probe were analyzed in 1 mm steps^26^. The corridor’s dimensions were defined to allow screw positioning if that bore probe with a diameter of 7.5 mm would not affect surrounding cortical bone along the corridor except for the entry and exit points. Affecting cortical bone was defined by exceeding a threshold of 400 HU^27–29^. This calculation was performed on the overall mean model and on both pubic ramus of all bone models resulting in a total of 300 individual bore probes available for evaluation. In case of perforation of cortical bone, except for the entry and exit points, the screw corridor was judged as not accessible and the sample was excluded from length analysis. In all samples with an available screw corridor, screw length was measured. For further evaluation of the local bone stock, the pattern of bone stock distribution of every single sample with an available screw corridor was normalized to the average screw length and graphically visualized.

The excluded samples were analyzed regarding the exact localization of undesired cortical perforation and categorized in three groups: perforation along the superior pubic ramus (group 1), perforation of the acetabular joint surface (group 2), perforation along the superior pubic ramus and of the acetabular joint surface (group 3).

Statistical analysis was performed applying Mann–Whitney-U test for independent samples and Pearsons’s Chi-Square analysis using the software SPSS (IBM SPSS Statistics 24, IBM, Armonk, NY, USA). Level of significance was set to a = 0.05.

### Ethical approval

All methods were carried out in accordance with the relevant guidelines and regulations. CT data were used retrospectively and anonymized with patient’s informed consent and approval of the local ethics committee (Ethikkommission Bundesland Salzburg, Austria).

## Results

A transpubic corridor with a diameter of ≥ 7.5 mm was available in 185 of 300 investigated superior pubic rami (61.7%; 110 male (67.9%), 75 female (54.3%)). Pearsons’s Chi-Square analysis showed no statistically significant relation between sex and availability of screw corridor (Chi-Square(1) = 7.205; p = 0.10). Mean length of the corridor was measured with 131.7 mm (standard deviation ± 10.7 mm). Mean length in the male samples was 131.5 mm (± 10.2 mm), and 132.1 mm (± 11.5 mm) in the female samples. No relevant sex-related difference in screw length was observed with p = 0.651. With respect to ethnicity, no statistically significant difference was shown neither regarding availability of the screw corridor nor the screw length with a mean screw length of 131.6 mm in Europeans and 131.9 mm in Japanese (p = 0.844) (Table 1).

**Table 1:** Statistical overview of the screw length.

Screw length		Mean (mm)	Minimum (mm)	Maximum (mm)	Standard deviation	p-value
All samples (n = 185)		131.7	104.9	161.4	10.71	
Sex	m (n = 110)	131.5	109.8	160.3	10.22	0.651
	f (n = 75)	132.1	104.9	161.4	11.45	
Ethnicity	Europeans	131.6	104.9	160.3	10.23	0.844
	Japanese	131.9	108.1	161.4	11.65	

Screw length shows a consistent range also comparing subgroups (sex and ethnicity). Statistical analysis was performed using Mann–Whitney-U-Test for independent samples and confirmed the observation that there is no significant difference between the groups.

**Figure 3:**
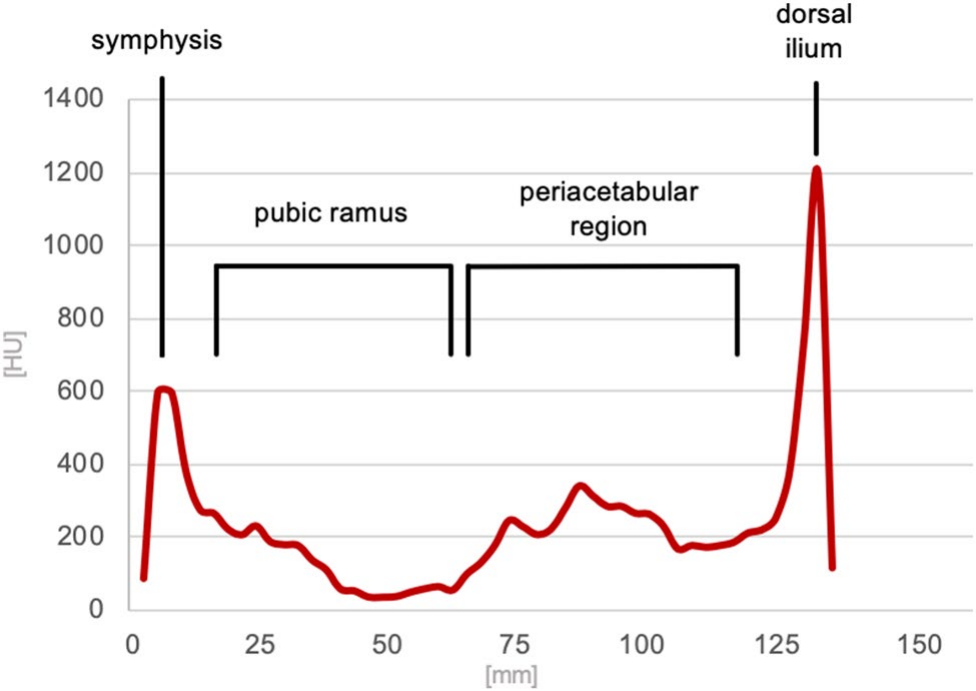
Mean local grey value distribution measured along the screw corridor showing a first cortical peak at the entry point, lower HU in the superior pubic ramus, slightly higher grey values in the periacetabular trabecular bone and a strong second cortical peak finally passing the cortex of the lateral ilium.

The local grey value distribution of the included samples showed a consistent pattern with a peak at the first cortex perforation in the area of pubic tubercle. Low HU values were seen along the superior pubic ramus, while higher values with an intermediate peak were present in the subchondral bone around the acetabulum with at least a peak of high HU values passing the cortical bone of the lateral ilium. Figure 3 displays the grey value distribution along the screw corridor.

**Figure 4:**
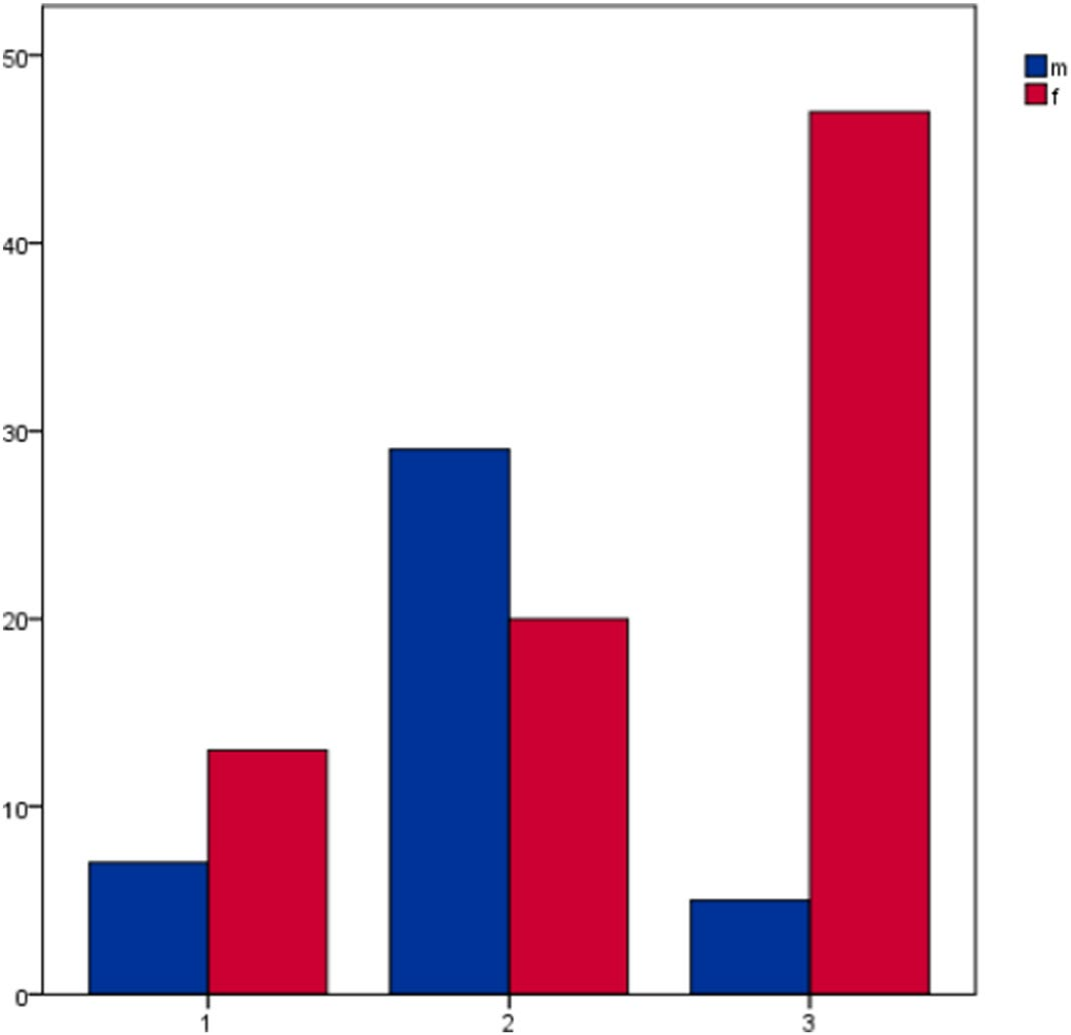
Samples with undesired cortical screw perforation, the figure shows distribution across the three defined zones in relation to sex; x-axis shows the area of cortical perforation (group 1 = perforation along the pubic ramus; group 2 = perforation of the acetabular joint surface; group 3 = perforation along the pubic ramus and the acetabular joint surface), y-axis shows the number of samples.

Analysis of the excluded samples showed that cortical perforation occurred predominantly at the acetabulum (group 2) in the male samples while in females the perforation was observed to be more often located at the pubic ramus and the acetabulum (group 3). Figure 4 and Table 2 show the distribution of the perforation site.

**Table 2:** Excluded samples with undesired cortical perforation, group 1 (pubic ramus), group 2 (acetabular joint surface) and group 3 (pubic ramus and acetabular joint surface) in relation to sexes.

Cortical perforation	Pubic ramus	Acetabulum	Pubic ramus and acetabulum
Male (n = 41)	7	29	5
Female (n = 80)	13	20	47

## Discussion

In the present study, almost two thirds of individuals have sufficiently sized transpubic corridors to position a retro- or antegrade transpubic screw. There was no difference in sex or ethnicity concerning length of the corridor. Intermediate grey values were observed in the subchondral region adjacent to the acetabulum, and high grey values in the lateral cortex of the ilium.

Retrograde transpubic screw fixation is a frequently used method to treat fractures of the anterior pelvic ring^30^. Several studies have investigated the screw corridor. Suzuki et al. performed a CT-based evaluation of the screw corridor on 160 CT data sets regarding length and diameter of the corridor. They found a mean length of the corridor of 124.6 mm in men and 123.8 mm in women. Minimal diameters were 13.5 mm in men and 10.7 mm in women in this study^20^. In contrast, Attias et al. published in a study on 13 individual, CT-based computer models a mean maximum screw length of 173.5 mm and a mean maximum virtual diameter for a potential implant of 6.4 mm^31^. Puchwein et al. investigated the corridor length and diameter in 50 CT polytrauma scans (35 male, 15 female) without pelvic injuries. The corridor was always available with a mean length of 127.2 mm and mean minimal diameter of 14.6 mm^32^.

Several biomechanical studies compared retrograde screw osteosynthesis with plate osteosynthesis. Simonian et al. showed that with a 4.5 mm retrograde transpubic screw, stability is similar to a conventional 3.5 mm plate osteosynthesis^33^. Acklin et al. compared a 7.3 mm screw osteosynthesis with ten-hole plate osteosynthesis. By using six of the ten possible screw options, a significantly higher stability of the plate osteosynthesis was shown under cyclic loading^17^. Another study of the same group showed a similar stability for a 7.3 mm screw compared to two 3.5 mm screws in the same biomechanical test setup^16^.

The present study shows consistent results in measurements of the length of a retrograde transpubic screw in adult pelves without relevant sex- or ethnicity-related differences. In the literature, screw length is reported at significant variability with mean screw length measurements, i.e. between 124 mm^20^ and 174 mm^31^. In our study, we included only those pelves which allowed for the positioning of a screw reaching the cortex of the lateral ilium without cortical affection of the bone except from entry and exit point. Consequently, shorter screws ending medial to the acetabulum were excluded from analysis. This might have resulted in the observation of a relatively higher screw length as reported in other studies^20,32^.

Although there was no sex-related difference of the screw length observed, we could show that screw positioning was less frequently possible in females than in males. This might be related to a narrower corridor with greater curvature due to sex-related anatomical characteristics^24^. For the same anatomical reasons, the site of undesired cortical perforation in the excluded samples appears to be sex-related.

Analyzing the grey value distribution following the screw corridor, we observed the strongest bone, as expected, on site of the lateral ilium. But the analysis showed also higher HU in the trabecular bone of the periacetabular region. We conclude that, at least the periacetabular region should be reached with the screw thread to achieve a sufficient anchoring, even if the positioning of a bicortical screw is not possible without affecting the acetabular joint surface due to the individual anatomical configuration. The results of the grey value analysis suggest that a retrograde transpubic screw fixation might provide a higher stability than an antegrade fixation due to the strong cortical bone of the lateral ilium and therefore more sufficient anchoring of the screw thread. A biomechanical study on synthetic bone models by Osterhoff et al. showed no differences regarding construct survival and stability between ante- and retrograde transpubic screw fixation^34^. According to our results, biomechanical testing on human bones is needed for further investigation due to the uneven bone mass distribution with relevant differences in quality and thickness of both, the cancellous and the cortical bone over the pelvis which can be not fully reproduced with the synthetic bone model.

As a limitation of the study it must be mentioned that the investigation was performed on uninjured pelvis. In treatment of fractures of the anterior pelvic ring, an exact anatomical reduction is not always necessary. Consequently, the accessibility of the screw corridor might be positively influenced by the fracture itself allowing slightly more flexibility for screw placement. Our results might therefore report a rather pessimistic number of possible transpubic screws. Further, also the usage of screws with a smaller diameter has to be discussed as a smaller diameter might increase the accessibility of the screw corridor. In our study, we used a virtual screw with a diameter of 7.3 mm as this is the implant we use in daily clinical practice for transpubic screw positioning with good clinical^1^ and biomechanical^16,17^ results available.

In conclusion, the present study shows that positioning of a transpubic screw was possible in almost two thirds of the examined samples. Possibility of screw positioning depends on individual anatomical characteristics determining the dimensions of the screw corridor. Therefore, transpubic screw fixation has to be planned carefully preoperatively with respect to the patient’s individual anatomy. The highest HU values were found, beside the cortex of the lateral ilium, in the trabecular bone of the periacetabular region which makes us assume that screws anchored with the thread in those regions might provide the highest stability.
